# Childcare for farm families: A key strategy to keep children safe yet largely absent from farm programming

**DOI:** 10.3389/fpubh.2022.1043774

**Published:** 2022-11-08

**Authors:** Florence Becot, Shoshanah Inwood, Andrea Rissing

**Affiliations:** ^1^National Farm Medicine Center, Marshfield Clinic Research Institute, Marshfield, WI, United States; ^2^School of Environment and Natural Resources, The Ohio State University, Wooster, OH, United States; ^3^School of Sustainability, Arizona State University, Tempe, AZ, United States

**Keywords:** farm business, farm safety, children, childcare, farm programs and resources, farm organizations, farm women

## Abstract

Despite long-standing safety recommendations that non-working children be supervised off the worksite by an adult, little is known about farm families' ability to comply. We conducted a review of 92 documents and 36 key informant interviews in three U.S. states (Ohio, Vermont, and Wisconsin) to assess how farm service providers and farm organizations address the intersection of children and childcare with farm work and farm safety in programming. Through their programming, these two groups deeply influence farm families' social systems, affecting farm safety and farm business decisions. Study design and result interpretations were grounded in the women in agriculture literature, which examines the needs and realities of farm women (often the primary caregivers). Most documents reviewed did not address children, and even fewer addressed childcare. Interviews confirm findings of the document review. Despite awareness that farm families juggle work and children, few interviewees explicitly integrated children and childcare topics due to a messy and complex set of individual- and structural-level factors. We identified four possible, overlapping explanations for this tension: valuation of care vs. farm work; farm programming's traditional emphasis on the farm business; alignment of the programming with the agrarian ideal of the family farm; and the mismatch between farm programming scope, resources available, and childcare challenges. We conclude with two main implications for farm safety programs and farm children safety. First, farm programming's reinforcement of the social and cultural expectations regarding children's involvement in the farm operation from a young age could be counterproductive from a farm safety standpoint and miss an opportunity to provide alternative models of childrearing. Second, the invisibility of the lived realities of raising children may lead farm parents to distrust farm programming and deter them from participating.

## Introduction

Farm safety researchers and educators have worked for decades to reduce the high rates of injuries and fatalities experienced by farm children. Despite the progress made as a result of these efforts, some have argued that injuries and fatalities rates remain too high, in particular among by-standing non-working children (1, 2). One explanation is the regulatory framework where no laws prevent farm parents from involving their children in the farm or the existing laws are deemed insufficient and/or not enforced (3–5). Another explanation is the low uptake of recommended safety strategies by farm parents (6–8). A key strategy to limit risk exposure for non-working by-standing children is the supervision of children by a dedicated adult off the worksite (hereafter “childcare,” whether paid or unpaid) (9–12). Yet, research has found that farm parents in a range of countries continue to bring their children to the worksite even when aware of the risks (13–16).

Currently, research has provided insights into farm parents' and farm children's safety knowledge and behaviors (17–19), as well as the cultural and social motivations behind why farm parents bring their children to the worksite (13–15, 20). However, despite decades of farm safety interventions to encourage farm parents to limit farm children's access to the worksite, there exists a dearth of research on childcare in agriculture. We know of only four peer-reviewed studies on childcare and farm safety, all from the U.S. and all based on small sample sizes. Three focused on migrant farm worker parents (21–23), and one focused on farm owners/operators (24). Findings from Gallagher (1) and Hartling et al. (25) indicate insufficient research has assessed the efficacy of farm safety interventions aimed at improving the safety outcomes of non-working by-standing children, further underscoring the need to probe more deeply into the reasons that shape farm parents' use of childcare.

Outside the farm safety scholarship, research examining the persistence of family farms (i.e., their ability to stay on the land despite on-going changes) has found that access to affordable quality childcare is a common challenge among farm families in the U.S. and directly affects farm enterprise development along with farm family and farmworker relationships (26, 27). Meanwhile studies on women in agriculture in Germany (28), Nigeria (29), Scotland (30), Switzerland (31, 32), and the U.S. (33–35) have pointed to women's challenges in taking care of the children and to the lack of support. Such childcare challenges in the context of farm business indicate a need for expanding farm safety research by looking at how larger systemic and structural issues shape farm parents' safety decisions along with their children's safety outcomes. These questions are particularly essential to answer since COVID-19 both likely increased the presence of children on farms (24, 36) and heightened existing challenges in the childcare sector in the United States (37) and also in countries with traditionally stronger supports for families such as France (38), the United Kingdom (39), and Australia (40, 41). These questions also connect to the call from Lee et al. (42) to shift away from a focus on individual-level factors by adopting a systems approach that deepens our understanding of farm children safety outcomes.

In this article, we contribute to the understanding of farm parents' safety decisions by assessing how farm service providers and farm organizations address the intersection of children and childcare with farm work and farm safety in their programs and resources. Part of a larger research project to understand the links between childcare and farm children safety (43), our rationale is grounded in the Socio-ecological model (SEM) and thus first focuses on the larger environment before turning our attention to farm parents. The SEM, a well-established framework in the fields of human development and public health, examines the complex social systems in which individuals are embedded (also referred to as spheres of influence), and the ways in which individual's behaviors are shaped by these social systems (44, 45). Both farm service providers and farm organizations create and deliver programs, a key function within, and strong influence over, a farm family's social systems, business, and dynamics (42). It is therefore critical to assess how those providing technical assistance and how those representing the interests of farmers to a range of stakeholders understand and approach topics connected to children and childcare if we are to understand the safety decisions that farm parents make. Through a document review of 92 farm programs and resources identified through an environmental scan and 36 interviews with farm safety and business service providers along with farm organization representatives in three U.S. states (Ohio, Vermont, and Wisconsin), we answer the following research questions: (1) How do farm service providers and farm organizations integrate topics connected to children and childcare in their programming? (2) What are farm service providers and farm organizations' perspectives on the interactions between children, farm business, and farm safety? and (3) What factors shape the integration of children and childcare topics in programs and resources? Farm programs and resources (hereafter farm programming) include the tangible and non-tangible educational outreach developed and/or deployed by farm service providers and farm organizations which include, for example, fact sheets, articles, tools, workshops, trainings, one-on-one service delivery, and initiatives. We consider children and childcare to be comprehensive of both the population of focus and activities connected to caring for that population of focus.

Given the farm safety knowledge gaps around childcare use, and given that women continue to play a primary role in caring for the children including in agriculture (27, 46, 47), we grounded our research design and interpretation of the findings in the women in agriculture literature [for reviews of the literature see: Ball (47), Brandth (48), Dunne et al. (49, 50)]. In particular, we draw on a line of research on farm programming and the representation of women in farm organization in Western industrialized countries which, for the English-language literature, is largely from Australia, the U.K., and the U.S. Despite variations in the social, cultural, political, and economic systems across these countries, this body of work provides similar key insights around the inclusion of women's needs and lived realities in farm programs and farm organizations. In addition, this body of work provides sociological insights around gender roles and division of labor, recognition and valuation of different types of work (i.e., care work, house work, farm work), and the reproduction of social norms and structures. See for example Shortall (51), Shortall (52), Shortall and Adesugba (53), Pini (54), Liepins and Schick (55), Alston (56), Alston (57), Trauger et al. (58), Trauger et al. (59) whose work we will discuss further when interpreting our findings.

Our article contributes to the farm safety literature by expanding the field in at least two ways. First, we move beyond the current focus on the individual-level factors that shape farm safety decisions by foregrounding the contexts in which these decisions are made. Recognizing that farm parents are informed by a complex landscape of farm programs, we consider the programming from farm business service providers and farm organizations which is an important expansion of the farm safety evaluation research that has traditionally focused on farm safety programs [see for example: Gallagher (1), Rautiainen et al. (60)]. Second, we bridge the farm safety literature and the women in agriculture literature which, despite many overlapping focal themes, have largely remained siloed. The farm safety field has recently called on the need to consider the specific needs women (61) and the interactions between farm children safety and farmwomen wellbeing (24). Developed by rural social scientists around the world starting in the 1970s−1980s, the women in agriculture literature provides an extensive body of knowledge around the social, economic, and cultural conditions of farm women; all key to understanding farm family dynamics, farm safety and wellbeing outcomes.

## Methods and analytical strategy

We used a two-steps mixed-methods research design to answer our research questions. First, we draw on secondary data from an environmental scan of publicly available farm programming. Second, we draw on primary data from key informant interviews. From a methodological standpoint, the merging of these two approaches, which we describe in detail below, enables the development of broad and nuanced insights to answer the research questions (62, 63). Furthermore, the triangulation of data of different types and sources is an important strategy to assess the validity of the findings (62, 64, 65). From a conceptual standpoint, our merging of public material and original interview data was informed by Liepins and Schick (55), who proposed a framework to critically analyze agricultural training as it pertains to meeting the need of women in agriculture. Expanding on the work of Shortall (51) who had drawn on the sociology of education and drawing on Foucault (66), a prominent scholar of power and knowledge, Liepins and Schick (55) argue that an analysis of farm programming content and the discourse around that content provides key insight around whose needs are served, what and whose knowledge is seen as legitimate, and who holds power in society.

As part of a larger research project, the three study states (Ohio, Vermont, and Wisconsin) were selected to capture: (1) the family farm model, a historically prominent farm structure in the Midwest and Northeast (67), (2) variations in farm commodity and scale (dairy in WI and VT of different sizes, large commodity crop production in OH, and smaller diversified operations in VT) (68), (3) variations in childcare environments with VT providing an extreme (or deviant) case study site (63) due to the state's significant investments in early childhood education prior to COVID-19 (69, 70). In addition, researchers had existing professional networks in these states along with on-the-ground knowledge of the agricultural sectors. The environmental scan is based on publically available secondary data and did not require a review by our Institutional Review Boards (IRBs). The IRBs determined the key informant interviews exempt from review.

### Document review of farm programming

We collected the farm programming material through an online environmental scan conducted between November 30 and December 21, 2020. We used a set of search terms including “farm safety,” “beginning farmer,” “young farmer,” and “women in agriculture” in combination with the names of our study states “Ohio,” “Vermont,” “Wisconsin.” While farm safety is an important aspect of this research, we used the three other search terms to identify the broad range of farm programming that are likely to reach families with younger children. This approach is in line with the SEM to understand the landscape of institutional organizations in which farm parents make decisions connected to their children (42, 44, 45). Because our focus was on understanding how prevalent topics related to farm children and childcare are within the broad range of programming targeted at farmers, we did not include “children” and “childcare” as initial search terms. We used two search engines to reduce search bias and increase reliability: Google and DuckDuckGo (a search engine that does not customize results based on previous searches) and conducted the searches until reaching saturation (i.e., no new relevant programming were found). We screened the 194 identified search findings and removed 102 that were either duplicates or non-relevant programs/resources (i.e., non-U.S. programs, previous events). The searches were originally focused geographically on our three study states. However, our search led to the identification of regional and national programming and we elected to retain these search results since their programming are accessible to farmers in our study states. We recorded the following information into a spreadsheet for each of the 92 remaining search findings: organization and program/resource name; search type (i.e., which keyword led to the identification of the program/resource); geographical area of focus (i.e., OH, VT, WI, national, regional); and contact information for key informant interviews recruitment. We also saved PDFs of programming (i.e., we electronically printed relevant sections of websites and downloaded relevant attachments found on websites).

We conducted two rounds of data coding and analysis on the 92 search results. First, we reviewed each of the search results and categorized them using a codebook developed based on the study goals and observations of the search results. There sets of codes were applied to both text and pictures. The first set of codes documents whether the resource/program targets a specific population of farmers (i.e., young farmers, beginning farmers, women farmers, farm employers) based on a yes (1)/no (0) scoring. The second set of codes determines the focus of the program (i.e., farm business, farm safety, balancing work/life), also based on a yes (1)/no (0) scoring. The third set of codes asked to what extent children/family and childcare aspects are incorporated into the farm programming based on a 3-point ranking to document the offering's continuum: (1) No mention; (2) Inconsequential mentions for material with broad statements and/or pictures but no practical information or resources. Examples of inconsequential mentions include a statement about the value of raising children on the farm or a statement about the importance of keeping children safe; and (3) Integrated in programming for content with practical guidance or resources to navigate children on the farm and/or to ease childcare access. Examples of integrated programming include a farm business planning worksheet with a section on the role of all household members including childcare duties, childcare/school expenses line in a budget tool, or advice to consider family needs and community amenities when purchasing farmland.

We conducted univariate analysis to assess the frequency of each code. In the second round of data coding and analysis, we qualitatively assessed the content of the programming by taking notes on our observations of text and pictures. We focused on describing the incorporation of children/family and childcare into the material across the three main program/resource focus (i.e., farm business, farm safety, balancing work/life). We then reviewed and summarized the content of these notes. Since we reviewed the material from most organizations included in our key informants sample, this article does not include visual examples (e.g., screen shots) in order to preserve the anonymity of the interviewees per our IRB protocol.

### Key informant interviews

Key informants included farm safety service providers, farm business service providers, and representatives of farm organizations. We identified these informants through: (1) project advisory board recommendations, (2) collated list of organizations and contact information identified through the document review, (3) the research team members' professional networks, and (4) a snowball sampling approach wherein we asked interviewees about other informants to interview (63). The goal of this multi-pronged approach was two-fold. The first was to limit selection bias by identifying interviewees through several avenues. The second goal was ensure a large enough list of potential interviewees to reach our target of 30 informants (10 per study states), a commonly accepted threshold to reach saturation (71–73). Our list of 85 potential interviewees included, among others, U.S. Department of Agriculture (USDA) young and beginning farmers' state coordinators, state departments of agriculture, University extension educators, and farm organizations. Out of the 60 individuals contacted, we interviewed 36. Seven declined to be interviewed most often citing that their programming does not incorporate children or childcare.

Key informants were interviewed with a semi-structured interview guide with branching questions that touched on five themes: (1) background information, (2) coverage of family, children, childcare topics, (3) childcare service offerings during programming, (4) childcare arrangements of the farm families they serve, and (5) the landscape of childcare in their geographical area. The branching questions were targeted to key informants working either on farm safety topics or farm business topics. To refine the interview guide's clarity, completeness, and flow, we sought feedback from the six-person project advisory board, comprised of a farm organization representative, a federal agency employee, professionals who work in farm business and farm safety topics, and a child development researcher—half of whom also operate a farm. We piloted the interview guide with two individuals in similar roles as targeted informants but outside of our study area, and revised the guide based on feedback. We conducted the interviews through the video conferencing platform, Zoom, and used the cloud recording function to generate auto-transcripts. Interviews lasted on average 52 min, ranging from 27 min to 1.4 h. A research assistant reviewed transcripts to ensure completeness and accuracy and to anonymize them.

Our final sample included 36 key informants across the three study states. On average, interviewed individuals were 46.7 years old, 83% of them were female, and 72% had direct personal connection to agriculture by growing up on a farm and/or working on a farm as an adult. Half of the respondents had a master's degree while over a third had a bachelor's degree and 14% had a doctoral degree. Looking at interviewees' organizational affiliation and focus area, over half (56%) were farm business service providers in organizations, while respectively 22% were farm safety service providers and from farm organizations. Given the limited number of potential interviewees in some organizational affiliations, we created categories that capture both the focus of the interviewee's work and their organizational affiliation to limit breach of confidentiality. Furthermore, these categories were productive to identify patterns in the data. The service provider categories included outreach professional and researchers with an outreach appointment in university cooperative extension services, non-profit organizations, or state or federal-level government agencies. Farm organizations are membership-based organizations that cover a broad range of farm-related activities. Last interviewees' state of residence was split almost evenly between Wisconsin (36%), Vermont (33%), and Ohio (31%). See Table 1 for interviewee's characteristics.

**Table 1:** Characteristics of interviewees (*n* = 36).

	***n***	**Proportion**
Average age in years	46.7	
Gender		
Female	30	83.3%
Male	6	16.7%
Personal connection to agriculture^*a*^		
Direct	26	72.2%
Indirect	4	11.1%
No	6	16.7%
Educational attainment		
Bachelors	12	33.3%
Masters	18	50.0%
PhD	5	13.9%
Other	1	2.8%
Organizational affiliations and focus areas		
Farm safety service provider	8	22.2%
Farm business service provider	20	55.6%
Farm organization	8	22.2%
State of residence		
Ohio	11	30.6%
Vermont	12	33.3%
Wisconsin	13	36.1%

^*a*^A direct connection to agriculture is when people reported having grown up on farms and/or having done farm work at one point or another. An indirect connection to agriculture is when people indicated a family background such as grand-parents operating a farm but no clear direct involvement in farm work.

We used a directed content analysis approach (74, 75), an iterative approach to qualitative data coding and analysis that includes deductive and inductive codes. The deductive codes were based on the interview guide questions (i.e., structural codes). The following is an example of a structural code group “Coverage of children/family and childcare aspects in programming” with the following sub-codes “Programing offered,” “No programming offered,” “Children/childcare programming,” “Farm parents' engagement,” and “COVID-19 related changes.” The inductive codes covered aspects of the data not covered through the structural codes but connected to the larger research aims and arising from the data (i.e., content codes). The following is an example of content code group “Children on farms” with the sub-groups “Intersection of children with farm business,” and “Intersection of children with farm safety.” The first and third authors coded and analyzed the data using the NVivo software (QSR International, Burlington, MA). To refine the codebook and to ensure consistency in use, we coded the same transcript, then used the Kappa co-efficient to discuss necessary codebook changes and coding approaches variations. We repeated this iterative process of two people coding the same transcript and addressing differences one time which is when we reached: (1) the average Kappa score of 75% across all codes, the threshold for excellent agreement (76) and (2) we were satisfied with usability and coverage of the codebook. We then split the remaining transcripts between the two coders and met on a regular basis to discuss potential adjustments to the codebook along with emerging themes and patterns. Upon completion of coding, the first author extracted and conducted a thematic analysis of the content of all codes related to the guiding research. The first and third authors regularly conferred to ensure identified themes and patterns were reasonable conclusions to draw from this body of data. Specific analytic attention was given to identify the range of responses and patterns in how these responses varied across main focus of program areas, organizational affiliations, and geography. The thematic analysis revealed organizational affiliation to be a more important factor than geography in explaining whether participants included childcare in their programming. The relatively small number of farm organizations in each state also makes maintaining anonymity more difficult when reporting state-specific findings. For both of these reasons, results do not name participants' states. To support the process of triangulation, we present the findings of the document review and key informant interviews together.

## Results

### Limited coverage of children and childcare topics in farm programming

The document review provides an overview of the integration of topics connected to children and childcare in farm programming. Out of the 92 program materials we analyzed, 53% made no mention of children/family topics and 83% made no mention of childcare. Almost one-third (29%) of the material reviewed included an inconsequential mention of children/family aspects and 12% of programming included inconsequential mentions of childcare (Table 2). Common examples of inconsequential mentions included pictures of smiling families with young children, statements about the value of raising children on the farm, or a statement about the importance of supervising children off the worksite to keep them safe. Last, 18% of the reviewed material integrated children/family in their programming while 4% of programming integrated childcare-related aspects. Two examples of the integration into programming included one business planning worksheet with a section inviting farmers to list the role of all family members including who is looking after the children plus an article outlining strategies to identify childcare (paid and unpaid).

**Table 2:** Coverage of children/family and childcare aspects in farm programming (*n* = 92).

	**Children/family aspects**			**Childcare aspects**		
	**No mention**	**Inconsequential mention**	**Integrated in programming**	**No mention**	**Inconsequential mention**	**Integrated in programming**
All material reviewed	53%	29%	18%	83%	12%	4%
Variations by target population						
Young farmers	45%	27%	27%	82%	9%	9%
Farm employers	15%	31%	54%	62%	23%	15%
Women farmers	54%	31%	15%	85%	8%	8%
Beginning farmers	61%	29%	10%	88%	7%	5%
Variations by primary focus						
Work life balance	20%	50%	30%	60%	20%	20%
Farm safety	30%	30%	40%	70%	21%	9%
Farm business	65%	29%	7%	92%	5%	3%

We found variations of coverage based on intended target audiences. Almost half (47%) of the material targeted beginning farmers, followed by women farmers (29%), farm employers (14%), and young farmers (12%) (defined by the USDA as those under the age of 35). Meanwhile, 16% of the material did not target one of these target audience. The material targeted to farm employers was most likely to touch on children/family and childcare aspects: respectively 85 and 38% made at least an inconsequential mention of these aspects. The material targeted to beginning farmers was the least likely to touch on these aspects as respectively 39 and 12% made at least an inconsequential mention of children/family and childcare aspects.

Looking at the primary focus of the programming, 67% of the material reviewed was primarily focused on the farm business (i.e., farm financials, farm production, and land access), 37% on farm safety for children and adults, 11% on work/life balance (i.e., mental health and family relationships), and 11% on other topics (i.e., leadership skills, advocacy, and networking). An inconsequential mention or better was most likely to be made by materials which focused on work-life balance. We found that 80% of these materials made at least an inconsequential mention on children/family and 40% of them made at least such a mention of childcare topics. On the other hand, programming material focused on the farm business were the least likely to touch on children/family or childcare, at 36% and 8%, respectively, making an inconsequential mention or better.

The key informant findings align with those of the document review in the sense that informants reported limited coverage of children and childcare topics in their programming. Furthermore, we found variations in the level of coverage based on the organizational affiliation and focus of the three groups of interviewees: farm safety service providers (22% of interviewees), farm business service providers (56%), and farm organizations (22%). Farm safety service providers were the most likely to integrate topics connected to children and childcare in their programming with recommendations and trainings targeted to farm parents, youth, and employers. Most of the integration was for children-related topics with the provision of information about children's exposure to risk and the provision of practical guidance to remediate these risks. Examples of practical guidance included designing safe play areas on the farm, the safe operation of machinery (e.g., ATVs, tractors), and the assignments and supervision of age appropriate tasks. The coverage of childcare topics by farm safety service providers was largely inconsequential. While most of these interviewees talked about the importance of childcare to keep the children safe, none provided practical guidance that would support use of childcare such as how to identify childcare and financial support to pay for childcare.

Farm business service providers were the least likely to integrate children and childcare into their programming. However, the coverage significantly varied across the different program areas, if information was integrated either formally or informally, and varied across the organizational structure farm service providers are embedded in. Programs for beginning farmers and farm management programs commonly wove in children and childcare topics when deemed relevant, but did not present on these issues as stand-alone topics. For example, a program might invite farmers to reflect on family goals and values alongside setting farm goals, or invite participants to include household-level needs when budgeting health insurance, childcare, and school costs and determining roles on and off the farm. A few interviewees emphasized how they bring up children in informal discussions particularly when working with beginning farmers, both to set realistic expectations and to deflate what they felt are over-romanticized expectations of raising children on the farm. Interviewees focusing on women in agriculture and farm transitions programs were the least likely to touch on children and childcare topics. When they did, it was indirectly through programming on family relationships, communication, and managing households.

The ways in which interviewees working for farm organizations touched on children and childcare through the lenses of farm business, farm safety, and quality of life varied considerably and fell along a spectrum ranging from minimal and informal discussions to intentional and formal programming. Interviewees on one end of the continuum reported that the farm organization they represented did not explicitly integrate children and childcare topics in their programming. At the same time, these interviewees spoke to the importance of family farms to their membership-base and to having a thread of family issues running through their work. For example, interviewees talked about creating a family-friendly space with children at events while another talked about conferences where family relationships and business might intertwine. Further along the continuum, interviewees talked about the explicit integration of these topics through blog posts, conference sessions, and virtual focus groups. Their emphasis was on immediate support to farmers through peer-to-peer sharing of stories and resources while also normalizing discussions about the challenges of raising children on farms. For example, one interviewee talked about bringing up household expenses such as childcare and health insurance in discussions about farm budgets because she finds that farmers do not bring up the topic on their own. At the other end of the continuum, some interviewees talked about the need for long-term solutions and the need to explicitly integrate children and childcare topics through their advocacy work and coalition building with childcare advocacy groups. These interviewees described a range of specific policy solutions such as universal childcare, and curriculum solutions such as farm service provider training to encourage and enable programing that would include information about existing resources to help farmers access childcare.

Finally, a consistent finding among key informants who actively integrated children and childcare topics was that they were already doing so before COVID-19. Still, the pandemic reinforced the need for this work. For those not integrating children and childcare topics, some interviewees shared they had become more aware of the challenges faced by parents as a result of COVID-19. We note the interviews were conducted in the early months of the pandemic and the extent to which future programming has emerged or will emerge is uncertain. Indeed, when asked about whether the interviewees' organizations were currently or planning to develop programs, resources, or policies to incorporate children or childcare in their programming, 80% said that they were not.

### Nuanced and layered set of perspectives on how children interact with farm business, farm safety, and parents' wellbeing

Despite the limited formal integration of children and childcare topics into programming, this sample of key informants provide a nuanced and layered set of perspectives of how having children on the farm interacts with the farm business and farm safety. It ought to be noted, however, that many of the reflections indicative of this nuanced and layered set of perspectives took time to emerge in the interview and occurred despite some informants indicating that they were not sure if they should participate in the interviews given that their programming did not cover children and childcare topics. Furthermore, some key informants stated that the interview questions allowed them to reflect on these issues and make connections they had not previously explicitly realized. For example, a farm business service provider said “*I mean until we had this conversation now, and never really, honestly it*'*s interesting to think about it”* (interviewee #9) and a farm safety service provider shared “*I mean just having this conversation today will make me think about well, what do parents who have a child that want to come to a program do?*” (interviewee #5).

Reflecting on the interactions with the farm business, interviewees spoke about the day-to-day impacts children have on the farm, and how children can dictate what and how much work can be done. Interviewees honed in on how children slow work down and the need to find ways to keep children busy as illustrated by a farm organization representative “*When I talked to two farmers who are parents, you know, they can work so much more quickly without three little kids following them around to do everything*” (interviewee #27) and reinforced by a farm business service provider “*Your productivity is definitely going to be less I think. It*'*s just a balancing act of what you need to get done, and it is affecting your income to the point where it offsets paying for childcare”* (interviewee #7). Interviewees also talked about the mid- to long-term impact of children, sharing stories of farmers making changes to their business structure, market channels, labor allocation, and growth plans in order to adapt to children. For example, a farm business service provider shared the experience of one farm family: “*he*'*s got five kids and he watches those five kids all day plus milks cows twice a day, and he went for a while there he went down to milking once a day that way he could even take care of the kids easier”* (interviewee #33) and another farm business service provider shared “*For children that are very young in a lot of cases what you will see where one of the parents kind of pulls back a little bit from the farm business to do a little more of that childcare if they can you know again that*'*s a sacrifice to the business and can be hard*” (interviewee #26). The document review and some of the key informants pointed to the social and cultural expectations that farm children spend time on the farm with their parents. For example in the document review, this was seen with the smiling pictures of children with their parents in the worksite along with statements around the benefits of raising children on the farm. Interviewees discussed how they navigate these social and cultural expectations, at times willingly at times unwillingly, through their programming. Farm safety service providers in particular have the added challenge of needing to navigate push back from the farm sector when farm safety recommendations may be perceived as going against farm parents' ability to integrate their children in the farm operation. This farm safety service provider spoke to some of that tension: “*We are mandated to train and certify 12 year-old children to be able to drive tractors and operate machinery. That's if you're going to work for a non-family farm. It goes directly against the NAGCAT guidelines, or the child safety guidelines, especially at age 12. I know it depends on the horsepower of the tractor and stuff like that. There are disconnects from things that are happening here in the state, what we are legally being not only allowed to do but made to do in terms of certifying those kids that really don't qualify if you follow those guidelines directly. Ethically that's challenged me*” (interviewee #28).

Farm safety interviewees frequently pointed to children's habitual presence on the farm and the implications for the children's safety. A farm business service provider stated “*You are probably going to want at some point in your parenting career rely on external childcare of some shape or form because there*'*s going to be some times, where you have to give your business your undivided attention, and it would just be too distracting or dangerous to have especially really young kinds on the farm”* (interviewee #14). Interviewees, across focus areas either explicitly or implicitly hinted at the complex balancing act farm parents face between the work that needs to get done, the danger children are exposed to, the farm safety recommendations, and the childcare options. For example, a farm organization representative said: “*The world could go through a pandemic, and suddenly your kids are all home, and you*'*re expected to not only keep the dairy farm going or the veggie farm going, but you*'*re also expected to homeschool and keep your kids safe, and there*'*s no one else to help you, and yeah, it*'*s a pretty—it*'*s been a pretty startling reality, and obviously, this last year has been extreme circumstances, but as so many people have said, it*'*s shown the glaring holes in the social safety nets that we have for so, so many people”* (interviewee #16). Interviewees described the strategies farm parents use to reconcile the competing need to get farm work done while keeping their children safe. Specific practices that key informants shared included: teaching children safety, completing the most dangerous tasks based on who is available to look after the children or when children are occupied, slowing down the work to make sure children do not get into something dangerous, teaching children safety, and giving them age-appropriate tasks.

Two overlapping but distinct paradigms among the majority of interviewees were used to explain why farm parents expose their children to risk. The knowledge-deficit paradigm was dominant especially among farm safety service providers. Some of these interviewees talked about the lack or inadequate awareness of farm parents to dangers, in particular among older generations, and the importance of educational programming to remediate the lack of knowledge. A farm safety service provider illustrates this paradigm: “*And in all fairness, part of my experience with them [farmers] is that some of them are simply not aware of the dangers and aware of how truly dangerous this is. I know that even for myself before I started my job that I'm in I didn't realize just how dangerous being a farmer could be and how dangerous that work site is*” (interviewee #29). In contrast, farm business service providers and farm organization key informants were less likely to see farmers as not knowing. Rather, they were more likely to approach the topic from a material-deficit paradigm, where parents are aware of the dangers but that the farm safety recommendations, in particular of keeping children away from the worksite, are either not practical or not feasible largely due to childcare challenges. A farm business service provider explained: “*Yeah I think even with farm safety, and that might be questioned you've got coming up too, is they just don't have another option out there. As far as you know well the kids have to be with me because there isn't no other option, nobody else to watch them so they have to be with me and but I can't watch them all the time when I'm doing chores so we just do our best, you know”* (interviewee #25). This material-deficit explanation was echoed among some farm safety providers, who while their work was grounded in the knowledge-deficit paradigm, they also acknowledged that high childcare costs and inadequate childcare services make it difficult for farm parents to adopt their farm safety recommendations as illustrated by this farm service provider: “*When we do presentations and when we work with farmers, and when we work with a lot of organizations that are service providers, our recommendation is always childcare, for you know whether that's an actual childcare center whether that's having a neighbor watch your child it's getting the child into care and out of that worksite so that's always the first option. But, as we know, with all the challenges in rural areas that isn't always feasible, so the second best thing is to have a safe play area*” (interviewee #29).

While we did not explicitly ask about the interaction between children and farm parents' wellbeing, the key informants consistently brought this issue up. More specifically, interviewees spoke about how the emotional and material aspects of raising children (as they pertain to farm business and farm safety) can have negative impacts on farm parents' mental health. From an emotional perspective, some interviewees spoke about the constant worries and/or guilt parents have in regard to the idea that their children could get hurt. A farm organization representative explained: “*Especially when we're looking at the seasons when we're planting or harvesting or there's a lot of things happening on the farm, I think. it's always in the back of your mind how are we keeping the kids safe, which provides an extra stressor when you do have the kids with you on the farm and how do you ensure that everyone who enters the farm knows that there are kids there and that they need to be aware of that.”* (interviewee #27) while a farm business service provider reflected: “*I think the worst case scenario is your one of your children gets hurt on the farm or another child gets hurt on the farm and I, you know I have just seen that happen, and it is really, really a heavy lift for women to have to get over that because the guilt is overwhelming”* (interviewee #14). Others spoke about the consequences of the disconnect between the romanticized view of raising children on the farm and the difficult reality as illustrated by this farm organization representative: “*Where folks have an idea that they can do it all that they can make enough money to live and save the environment, and you know tend to their children and their family needs like just by doing this farming thing and the reality ends up being often that like either the mental piece doesn't work out or the tensions with actually having the time and the ability to provide for your family in terms of you know wellness and child care and things like that don't line up with the farming or what's best for the land or best for the environment, often doesn't make the economic return that drives the other systems*” (interviewee #1). From a material perspective, interviewees spoke about the stress of juggling personal and professional responsibilities and the lack of support parents have. A farm business service provider recollected: “*I actually had one extension employee who worked at a different county office that I supervised. She had a dairy and two kids that were kind of close in age, like my two boys were. She was really struggling with this work being at home and helping on the farm. I had lunch with her one day she was crying she didn't know what to do. Right so I'm getting her to those employee assistant resources that she needs I'm sharing my personal experience with her. She ended up taking a leave of absence from work to be at home because she was having issues with childcare”* (interviewee #11). This group of key informants shared how parents stress is compounded by the need to grow their farm business while constantly needing to adapt their farm business and care strategies based on the availability of childcare options; the age, number, and needs of children; and personal preferences. These challenges were largely seen as most acute for farm families with younger children, limited family support to look after the children, and lack of financial resources to pay for childcare.

### Messiness and complexity of the factors shaping the integration of children and childcare aspects in farm programming

The analysis revealed several factors explaining whether interviewees and their organizations integrate children and childcare topics into their program, and the degree to which they incorporate these topics. These factors include: the demographic characteristics of farmers the programming serves, the needs of farmers, the program's conceptualization of the agri-family system, the program's scope of work, the organizational structure, the available resources, and the interviewee's lived experience. These factors are presented in a simplified tabular form (Table 3). As the integration of children and childcare topics in farm programming was rarely explained by just one factor, the goal of the tabular presentation is to provide a simplified way of presenting the factors and patterns identified, over developing an absolute typology.

**Table 3:** Factors explaining the level of integration of children and childcare aspects in programming.

**Little/no integration**		**Explanatory factors**	**Integrated in programming**	
Older farmers (e.g., farm transition and retirement programming)—men (e.g., production oriented topics)	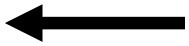	Farmers served by programming	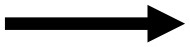	Younger farmers (e.g., beginning farmer programs)—Young kids (e.g., farm children safety programs)
Not aware that childcare is a problem—Don't know about childcare as a problem—Push back from farm sector on safety regulations—No data to determine if a problem	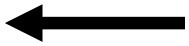	Farmers' needs	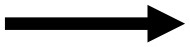	Aware that childcare is a problem—Challenges with childcare have worsened because of COVID-19
Household and business = separate entities: Business needs to pay for itself and interviewee want to limit the entanglement of the professional and personal spheres	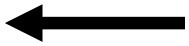	Conceptualization of agri-family system (i.e., interviewees' conceptualization of the relationship between the farm household and the farm business and the role each play)	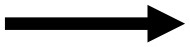	Household issues = business issues: holistic approach to the farm whereas household-level issues have direct implications on the farm business
Children/childcare aspects not connected to scope's of work—Childcare challenges bigger problems than what organization can offer	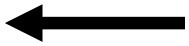	Program/organization's scope of work	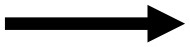	Grassroots nature of the organization and importance of addressing farmers' needs—Addressing childcare issues is connected to changing the narrative around how farmers are supported
Large and/or organization with top-down management limit ability to develop programming	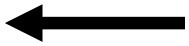	Organizational structure	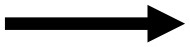	Small and/or organizations with flat hierarchy provide the space to react quickly and try innovative programs
No grants available to do this work—Decrease in capacity due to budget and staff shrinkages	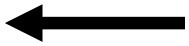	Availability of resources (time and money)	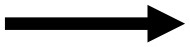	Farmer member-funding gives emphasis on responding to their needs—Use of COVID-19 relief funding to expand programming on childcare
No children—Wife providing childcare	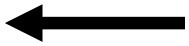	Lived realities and experiences of interviewees^*a*^	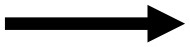	“I have been there”: first-hand experience with raising children, with childcare challenges

^*a*^ While our demographic mimetype="image" mime-subtype="tiff" questions were limited to farming background, age, and educational attainment, interviewees frequently volunteered information about their personal life providing key insights into their lived realities.

Given space limitations, we describe one explanatory factor in detail to illustrate how the table should be read: how demographics of the program's target farm population influence the integration of children and childcare in program development and deployment. Interviewees explained that programs tend to target specific farmer needs (e.g., farm transition or farm safety) and demographics (e.g., women farmers or young farmers). On one hand, among those interviewees least inclined to cover children and childcare in their programming were those delivering service to older farmers (i.e., farm transition and retirement) or those that tend to attract men (e.g., production oriented topics). On the other hand, interviewees most likely to integrate childcare and children tended to serve young farmers and farmers whose children were younger (e.g., beginning farmer and farm children safety programs). It is particularly striking that despite women continuing to serve as primary caregivers, programs targeting women in agriculture did not actively, intentionally or explicitly create or provide programming related to children and childcare.

We consistently found the reasons why children and childcare are or are not included in farm programming are the product of multiple interacting factors. For example, one farm safety service provider had raised their children on a working farm and spoke honestly about the challenges of finding childcare for themselves and other farmers. However, their program did not address childcare other than recommending farm parents arrange for adult supervision off the worksite. Reflecting on the reasons why their coverage of childcare was limited, they spoke to a tension (which we heard among other interviewees) wherein they struggled to reconcile their recognition that farm safety recommendations were financially and materially challenging for farmers, but at the same time felt childcare is a bigger issues than what their organization can address. The following quote from this interviewee speaks to the heart of this tension and further illustrates the complex and competing forces influencing program development and deployment. “*You know that's one of those messages that I say don't and then I hate when I have to say don't because is there an alternative that I can also give them rather than just saying don't do this. Remember, I talked earlier about self efficacy if you feel you can make a change, you probably will […]. If I say a parent don't just leave your kids you know don't bring them to your worksite well they will say I have to work and there's things to do so I guess, I have to just bring them. So there's certain things on the farm that it's hard as a safety professional to advocate for if I don't have a system or another alternative to offer them*” (interviewee #3).

## Discussion

In what follows, we first provide a summary of our findings to answer these three questions and discuss connections with the existing body of knowledge. As a reminder our research questions were: (1) How do farm service providers and farm organizations integrate topics connected to children and childcare in their programming? (2) What are farm service providers and farm organizations' perspectives on the interactions between children, farm business, and farm safety? and (3) What factors shape the integration of children and childcare topics in programming? Then, pointing to a key tension in our findings, we provide possible explanations around four interconnected themes. These are grounded in the women in agriculture literature on farm programming and farm organizations, the body of work that in part informed our study design.

### Summary of findings

In direct response to our first research questions, we found that, despite children being ubiquitous to family farms and despite childcare being a key farm safety strategy, the coverage of children and childcare aspects in farm programming was overall limited and largely inconsequential with an overall lack of practical guidance and resources to support farm parents in balancing the children, their safety, and farm work. This finding is not surprising. We noted the dearth of research on childcare for the agricultural sector in our introduction. In addition, Gallagher (1) and Hartling et al. (25) noted the lack of research evaluating farm safety interventions targeted to the youngest children while Inwood et al. (77) has noted the lack of attention to health insurance, another household-level issue, in the farm business programming. Within the landscape of the farm programming we assessed, those focusing on the farm business were more prevalent compared to those focusing on farm safety, yet it was farm safety programs that were more likely to touch on children topics while both farm business and farm safety programs seldom covered childcare topics. With the exception of a handful of farm organizations in our two samples that were actively working to provide practical in support of farm parents, the frequent emphasis on knowledge-deficit and behavior change paradigms in programming echo previous findings on farm safety (1, 2, 25, 60) and farm business (78–80) programming. Speaking to the second research question, the limited coverage cannot simply be explained by a lack of awareness among interviewed key informants given the nuanced and layered set of perspectives on how children interact with the farm business, farm safety, as well as farm parents' wellbeing. Interviewees' descriptions of the short and long-term implications of children on the farm business, strategies to keep the children safe, and stress associated with juggling multiple role and worrying about the children getting hurt echo those provided by farm parents (13, 24, 27). The layered and nuanced understanding of the material and economic realities of raising children on farms is likely due to both first hand-experience of some interviewees and the frequent interactions with farm parents for others. In response to the third research question, a messy and complex range of factors explained whether and how children and childcare topics were integrated in farm programming. Given that actions are the results of interconnected multiple spheres of influence (42, 44, 45), some of the factors were within the purview of interviewed individuals (e.g., their conceptualization of the agri-family system), while others were connected to factors outside their control as they pertained to factors within their organization (e.g., organizational structure) or within factors fully outside their organization (e.g., availability of resources).

In sum, our findings point to a key tension. On one hand, there is a general understanding among those developing and deploying farm programming that raising children on farms can be challenging with direct implications on the focus of their programming (i.e., farm safety and farm business). On the other hand, farm service providers and farm organizations are rarely integrating these topics in their work or, when they are, it is largely inconsequential and most often focused on changing farm parents' knowledge and behaviors. Our findings interpreted through the lens of central themes from the women in agriculture literature point to four possible interconnected explanations.

### Childcare work is not seen as farm work

Collectively and with variations among individual interviewees aside, our findings align with those of other researchers regarding how invisible practical, lived realities of raising children on farms can be. This includes lines of research on the invisibility of women's work (81, 82), farm women (31, 51, 83), and farm household-level issues (27, 84, 85). This also includes lines of research on the representation and integration of farm women in institutions pointing to women being underserved by educational programs (51, 55, 58, 59, 86), underrepresented in farm organizations (52, 53, 56, 87, 88), and their presence and contributions not included in farm statistics (54, 89, 90). The invisibility of women's work caring for children in comparison to the visibility of farm output work, traditionally seen as men's work, illustrate the undervaluation of women's work. This undervaluation of women's reproductive work over men's farm output production work has been discussed at length. See for example: (55, 57, 83, 91). We see evidence of this undervaluation in our data, even aside from the limited coverage of children and childcare topics. The initial reticence of some participants, citing not knowing about the topic, plus the self-reflections wherein some reported the interview helped them make new connections, speaks to the invisibility of childcare work on the farm.

### Farm programming is about supporting the productive function of the family farm

In line with the traditional emphasis of farm service providers and farm organizations, the farm programming we reviewed was largely about supporting the productive function of the family farm. The contours of what supporting the productive function entailed varied in part based on what is valued as farm work and what is not. Interviewees who perceived childcare as a problem, those who had first hand-experience raising children on farm and/or with childcare challenges, and those with a holistic conceptualization of the family farm (i.e., conceive that the personal and professional spheres overlap) were more likely to view the connection between children work and productive work. In turn, these interviewees were more likely to integrate children and childcare topics in their programming, be it formally or informally. While farm safety programs were primarily about the safety of children, their programming reflected the primacy of farm work over childcare work. Indeed, most of the farm safety programming was about keeping the children safe in the workplace while there was comparatively no practical programming to support childcare work. The limited coverage of children and childcare topics in programming targeted to women is on one hand surprising given that this programming is specifically designed to meet the needs of women. On the other hand, it could reflect farm women's desire for their professionalization and their identify as farmers in their own right (92) and support the finding that even women in agriculture programming has been found to not fully account for the complexity of their roles and needs (51, 58, 86).

The myopic focus on farm work topics, coupled with the limited coverage of childcare work in farm programming, connects back to the undervaluation of care work as noted above and illustrates which knowledge is seen as legitimate and which is not. The women in agriculture literature has previously noted both the oppressive and marginalizing nature of the farm programming and farm organizations for farm women, noting that which topics are covered and which are silenced reflect the preferred knowledge and prevailing power relations of a society (51, 55, 56, 58, 59, 87).

### Alignment of farm programming with the traditional family farm model

Some of our findings indicate an alignment between programming with the agrarian ideal of farming as a family affair. Such alignment may limit the space for farm programming that would suggest a move away from the prevailing social and cultural norms of raising children on the farm. Evidence of alignment with this agrarian ideal include pictures of smiling families with young children in the worksite and statement around the benefits of raising children on farms in the farm programming material and through the embodiment and reinforcement of the expectations that children are looked after by family members largely on the farm by key informants. These findings connect back to the women in agriculture literature which has highlighted the ways in which farm programming and farm organizations tend to adhere to family farm ideals which contributes to reinforcing traditional divisions of labor (51, 53, 56, 58, 59, 87). Potentially in play for farm safety service providers is a fear of push back from the agricultural sector given pushbacks in the past against a proposed major reform of child labor and safety in the early 2010s (93, 94). Indeed, farm safety material included frequent mentions of the benefits of raising children on farms in tandem with lack of practical programming on childcare.

### Mismatch between farm programming scope, resources available, and childcare challenges

A common pattern among interviewees was their discussion of factors connected to their larger environment as limiting the programming they can develop and deploy including mandated scope of work and access to resources. In particular, we found a constant push and pull between interviewees' perceived challenges, whether they believe they can respond to these challenges, and the context in which they work. As some interviewees noted, their programming is educational in nature, while the challenges of supporting a household on a farm income and/or using childcare are challenges that are structural in nature. This finding aligns with Calo (79) who found that prevalent knowledge-deficit approaches of young and beginning farmers on farm knowledge and skills fall short of addressing the structural nature of farm start-up challenges such as access to land and capital. The women in agriculture literature provides insights as to why those in leadership positions might not prioritize nor allocate funding for programs that would support farm parents, and working parents more broadly. Farm women, which as we noted above continue to be the primary caregivers, have historically been sidelined from leadership positions in farm organizations and political spheres. Given the important role of farm organizations in shaping the narrative for agricultural programming and policies, this means that decisions around which challenges should be addressed and how, along with how resources should be allocated have largely been made with little to no input from women and with little understanding of women's needs and realities (52–54, 56, 87, 88, 95).

### Limitations

Our findings and their implications need to be understood in light of three main limitations. First, in our choice of search terms, we selected search terms that would allow us to identify the broader landscape of farm programs and resources in which farm parents are embedded. We did not use “children” and “childcare” as search terms in the initial search phase. While this choice is a study limitation, we note that the findings from the key informant interviews align with those of the document review, indicating an appropriate coverage of our search terms. Second, our choice of qualitative methods coupled with a focus on three states was intended to develop an in-depth understanding of a topic that has received limited attention and to identify patterns. Our focus in this article was on the most prevalent patterns we identified in the data and as such do not fully speak to all of the information and perspectives shared by all informants. We recognize the need for further data analysis that explores in greater depth the margins of the patterns presented in this article. Furthermore, research assessing the same questions in other states is important to work toward the generalizability of findings. Our findings still likely provide important insights as we suspect that similar patterns to those we found will emerge. As noted in the methods section, our document review included national-level organizations. We also identified farm programming documents from national level clearing houses during our environmental scan. Though documents from states outside our study states were not included in our analysis, our informal review of these documents also suggested little to no coverage of children and childcare topics. Hinting to the potential to find similar patterns in other states, much of farm programming topics and funding priorities continue to come from the U.S. Department of Agriculture while state branches of large farm organizations tend to follow the programming and advocacy priorities set by the national-level organization. Last, childcare challenges as well as the undervaluation of care work are not unique to our three study states. Third, our positionality as female researchers, our prior work on this subject, or our affiliations may have biased responses provided by interviewees. We worked to minimize the bias though our interview introduction, non-leading questions and probes. Furthermore, during the coding and analysis phase, we had several conversations about the biases we might bring and ways to limit them.

## Conclusion

Farm safety experts have indicated that a reason for the continued high fatality rates among by-standing non-working farm children is in part explained by the low uptake by farm parents of recommended safety practices including the key strategy of adult supervision off the worksite (6–8). Yet the reasons why farm parents are using or not using childcare have received scant attention in the literature. The social systems in which farm parents make safety decisions for their children influence choices including childcare use. Thus, our analysis of 92 farm programming documents and 36 interviews with farm service providers and farm organization in three U.S. states assess how these actors address the intersection of children and childcare with farm work and farm safety. Overall, we identified a key tension in our findings whereas despite a layered and complex understanding of the challenges farm parents face juggling work and the children among most interviewees, few programs explicitly integrated children and childcare topics. Interviewees pointed to a complex and messy mix of individual and structural-level factors as shaping whether or not they integrated these topics in their work. Our interpretations, grounded in the women in agriculture literature, of this key tension led us to identify four possible and overlapping explanations around the valuation of care vs. farm work; the traditional emphasis of farm programming on the farm business; alignment of the programming with the agrarian ideal of the family farm; and the mismatch between farm programming scope, resources available, and childcare challenges.

Our findings in tandem with the theoretical and empirical insights from the women in agriculture literature raise concerns about the invisibility of children and childcare topics for farm programming, with all that such invisibility implies. Farm programs and farm organizations that do not adequately account for women's realities nor value their contributions deter women from participating and can reinforce societal norms around gender and divisions of care and farm work (51, 54, 87, 96). Social and cultural expectations around children growing up on farms and participating in farm work have been identified as a barrier to farm safety (13, 14, 16, 20). Yet the embodiment and reinforcement of these norms in farm programming could be counterproductive to achieving the goal of limiting children's exposure to risk because it implicitly indicate limited acceptance and space for farm parents who either cannot or do not want to adhere to these norms. Practical implications coming out of these concerns include hiring and retaining staff with a diversity of identities and lived experiences at all level of the organizations to re-think their programming while addressing organizational gender biases so that farm programs can reach a wider range of farmers on a wider range of topics (51, 55, 59, 96–98). The women in agriculture literature also points to the need to move away from top-down models of farm service provision and to instead move toward participatory programming models whereas farmers are seen as experts in their own right. One way to do that is by creating space for farmers to learn from one another (51, 58, 59). The practical realities of raising children on farms and implications for farm business and safety along with the provision of information on resources to ease childcare use need to be integrated in existing farm safety and farm business programming. To avoid reinforcing gender stereotypes and to make visible the realities of juggling children and farm work, it is crucial that the integration should not be limited to programs targeting women (52, 53, 56, 59, 87).

The mismatch between farm programming scope, resources available, and childcare challenges is harder to resolve given the structural factors at play around childcare access and cost. In turn, these findings reinforce the importance of multi-dimensional approaches as farm service providers alone cannot be expected to address complex problems (79). In the short-term and in-line with the farm organizations that most integrated children and childcare in their programming, farm service providers and farm organizations can augment their programming through collaborations with other groups working on childcare. This means collaborations with family and consumer education service providers (e.g., from extension services and state agencies of family services) and childcare advocacy groups to help connect farm families to childcare resources such as referral services and financial support. At the same time, farm service providers and farm organizations can serve these family and consumer service providers by recognizing that raising children on farms can be stressful and making sure that their programming accounts for the lived-realities of farm families such as non-traditional work hours, seasonality of the workload, cost, quality, and availability of childcare, and self-employed status (as it pertains to financial support eligibility criteria). In addition, farm organizations have a role to play by advocating with policy makers and departments of agriculture at the state and federal level for the broadening of farm programming so that it is inclusive of issues impacting the farm household over solely the farm business. In this advocacy work, farm organizations should collaborate with childcare advocacy groups as these groups already have experience and expertise addressing childcare challenges. At the same time, given that childcare advocacy groups tend to focus on urban areas, there are opportunities to build on the recent work from the U.S. Department of Agriculture and U.S. Department of Health & Human Services (99) on rural childcare initiatives by ensuring that the needs and realities of farm families are incorporated in this work.

Two lines of research to investigate emerge out of our study. The first line of research needs to tease out the adequacy of the four themes we identified to explain the key tension in our findings. Given that these themes were grounded in the women in agriculture literature, one approach to future research could be through a formal gender analysis of farm programming in the U.S. but also beyond the U.S. This could include an assessment of how farm service providers and farm organizations understand care work to overlap with or depart from farm work. The second line of research needs to assess the implications of the invisibility of children and childcare in farm programming. Most relevant to the farm safety field, this includes the need to understand the extent to which the reinforcement of the social and cultural norms of raising children on farms is limiting the use of childcare. Farm service providers might call on these social and cultural expectations to relate with the farm families. Yet and connected to the findings of Janssen and Nonnenmann (100) and Neufeld and Cinnamon (101), our findings raise questions about the lessening of trust in farm programming among farm parents when the programming does not account for their lived realities and stress of raising children on the farm. Our findings also raise questions around the consequences of reinforcing social and cultural norms around raising children on the farm and of not making space for alternative approaches on the use of childcare by farm parents.

## Data availability statement

The original contributions presented in the study are included in the article, further inquiries can be directed to the corresponding author.

## Ethics statement

The studies involving human participants were reviewed and approved by Marshfield Clinic Research Institute and The Ohio State University. Written informed consent for participation was not required for this study in accordance with the national legislation and the institutional requirements.

## Author contributions

FB: funding acquisition, project administration, conceptualization, data collection, data curation, data analysis, and writing—original draft and editing. SI: funding acquisition, conceptualization, data collection, and writing—review and editing. AR: conceptualization, data collection, data analysis, and writing—review and editing. All authors contributed to the article and approved the submitted version.

## Funding

This work was supported by the National Institute for Occupational Safety and Health under Grant U54 OH009568-10 and the Marshfield Clinic Research Institute.

## Conflict of interest

The authors declare that the research was conducted in the absence of any commercial or financial relationships that could be construed as a potential conflict of interest.

## Publisher's note

All claims expressed in this article are solely those of the authors and do not necessarily represent those of their affiliated organizations, or those of the publisher, the editors and the reviewers. Any product that may be evaluated in this article, or claim that may be made by its manufacturer, is not guaranteed or endorsed by the publisher.
